# Pathogenesis of eosinophilic chronic rhinosinusitis

**DOI:** 10.1186/s12950-016-0121-8

**Published:** 2016-04-06

**Authors:** Said Ahmad Shah, Hajime Ishinaga, Kazuhiko Takeuchi

**Affiliations:** Department of Otorhinolaryngology, Head and Neck Surgery, Mie University Graduate School of Medicine, 2-174 Edobashi, Tsu, Mie 514-8507 Japan

**Keywords:** Chronic rhinosinusitis, Eosinophilic chronic sinusitis, Eosinophils, Cytokines, Mucus, Diagnosis, Clinical features, Treatment

## Abstract

Eosinophilic chronic rhinosinusitis (ECRS) is considered a refractory and intractable disease. Patients with ECRS present with thick mucus production, long-term nasal congestion, loss of sense of smell, and intermittent acute exacerbations secondary to bacterial infections. Despite medical and surgical interventions, there is a high rate of recurrence with significant impairment to quality of life. The recent increasing prevalence of ECRS in south Asian countries and the strong tendency of ECRS to reoccur after surgery should be considered. The majority of cases need repeat surgery, and histological examinations of these cases show eosinophilic-dominant inflammation. The degradation and accumulation of eosinophils, release of cytokines, and mucus secretion have important roles in the pathogenesis of ECRS. ECRS differs from non-ECRS, in which eosinophils are not involved in the pathogenesis of the disease, and also in terms of many clinical characteristics, blood examination and nasal polyp histological findings, clinical features of the disease after surgery, efficacy of medications, and computed tomography findings. This review describes the clinical course, diagnosis, and treatment of ECRS as well as its pathophysiology and the role of eosinophils, mucus, cytokines, and other mediators in the pathogenesis of ECRS.

## Background

Eosinophilic chronic rhinosinusitis (ECRS) is an inflammatory pathological condition of the nose and paranasal sinuses [1]. Patients with ECRS present with loss of smell, long-term nasal congestion, thick mucus production, and intermittent acute exacerbation of secondary bacterial infections. The quality of life of these patients is severely impaired [2]. ECRS is a subtype of chronic sinusitis that is thought to occur secondarily to systemic eosinophil deregulation [3]. Patients with chronic rhinosinusitis (CRS) in the United States and Europe are classified into two subtypes: CRS with nasal polyps (CRSwNP) and CRS without nasal polyps (CRSsNP) [4, 5]. The majority of patients who have recurrence after surgery for nasal polyps have pronounced eosinophilic infiltration of nasal polyp tissue. Additionally, these patients have a strong tendency for the recurrence of nasal polyps after surgery [5]. In East Asia, most CRS patients exhibit purulent rhinorrhea including abundant neutrophils [6] or neutrophils together with fewer eosinophils [7]. These findings suggest that CRSwNP is heterogeneous and can be divided into two subtypes: ECRS and non-ECRS [8]. However, in Japan and other East Asian countries, such as Korea, less than 50 % of CRSwNP patients exhibit such eosinophilic-dominant inflammation, suggesting that the pathophysiological presentation of CRS differs by race, climate, and geographic region [9–11]. In short, ECRS is the major endotype of CRSwNP in the United States and Europe, and has been increasing in prevalence in Asia [12, 13]. From 1999 to 2011 in Thailand, there was a predominant change in patients from neutrophilic to eosinophilic CRSwNP [12]. In Japan, in 2001, the term ECRS was introduced to identify this subgroup of patients with rhinosinusitis and eosinophilic infiltration of nasal polyps [14]. Patients with ECRS represent a unique subtype, and they especially remain the most resistant to medical and surgical interventions [15]. Patients with ECRS show a strong possibility of overlapping mechanisms for eosinophilia and have a poor response to medical and surgical management. Therefore, ECRS is considered to be a refractory and intractable disease [2]. Several stimuli, including fungal antigens, allergens, bacteria, and bacteria-derived superantigens, may be involved in the pathophysiology of ECRS [16]. Thus, ECRS is thought to reflect an inflammatory process and encompasses a wide variety of etiologies [17]. The following subcategories provide adequate information and support mechanisms that involve eosinophil infiltration and inflammation in ECRS: 1) superantigen-induced ECRS [18], 2) allergic fungal sinusitis [19], 3) nonallergic fungal ECRS [20], and 4) aspirin-exacerbated ECRS. Within each subcategory, a specific antibacterial, antifungal, or immune process may be indicated [15]. However, substantial confusion exists especially in the categorization of fungus-related eosinophilic rhinosinusitis [3, 20]. Certainly, there are other mechanisms and categorizations of ECRS that are still unknown. In this review, we discuss and focus on treatment strategies and pathogenesis of ECRS from different angles including the role of eosinophils, cytokines, mucus production, and other mediators as well as explanations of its clinical course, diagnosis, and therapeutic interventions.

### Cytokines, mucus, and other mediators

Multiple cytokines are involved in the pathogenesis of ECRS. Elevated levels of circulatory eosinophils and tissue eosinophils are prominent features of ECRS. Cytokines are essential for hematopoietic cell development, differentiation, and maturation. Interleukin (IL)-3, IL-5, and granulocyte/macrophage-colony stimulating factor (GM-CSF) are cytokines that are particularly important in regulating eosinophil development [21]. Myeloid precursors are responsible for the formation of eosinophils in bone marrow in response to cytokine activation, and following an appropriate stimulus, they are released into the circulation [22]. IL-5 is essential for the maturation of eosinophils in the bone marrow and their release into the circulation [23–26]. Once eosinophils have entered the blood, they have a short half-life, ranging from 8 to 18 h [27]. After circulating in the blood, eosinophils migrate into tissues. The tissue life span of eosinophils ranges from 2 to 5 days [28]. Various cell types are responsible for the production of IL-5 [29]. In humans, IL-5 is a very selective cytokine for eosinophils and basophils, in which it promotes maturation, growth, activation, and survival [30, 31]. This specificity occurs because only those cells express the receptor for IL-5. Once eosinophils enter the circulation, they accumulate rapidly in tissues and synthesize and release lipid mediators, thereby causing edema, bronchoconstriction, and chemotaxis. Furthermore, eosinophils secrete enzymes and proteins that can damage tissues [22].

Eosinophils also function as antigen-presenting cells and they can process and present a variety of microbial, viral, and parasitic antigens [32]. In addition, eosinophils treated with GM-CSF promote T cell proliferation in response to staphylococcal superantigen (Staphylococcus enterotoxins A, B, and E) stimulation [33]. Furthermore, incubation of human rhinovirus-16 with eosinophils promotes rhinovirus-16-specific T cell proliferation and interferon (IFN)-γ secretion [34]. Platelet-derived growth factor receptor alpha (PDGFRα) is implicated in cell growth, transformation, proliferation, migration, and vascular permeability. Platelet-derived growth factor-α (PDGFα) is a specific ligand for PDGFRα. IL-4 together with IL-5, IL-1β, and PDGFα are critical for PDGFRα gene expression that play a pivotal role in the pathophysiology of ECRSwNP and non-ECRSwNP [35]. In a recent study, an anti-IL-9 antibody significantly reduced bone marrow eosinophilia in an animal model; IL-9 was over-expressed in bone marrow CD4+ cells after allergen exposure, suggesting that IL-9 may participate in the regulation of granulocytopoiesis in allergic inflammation [36]. IL-13 is a critical cytokine in the pathogenesis and development of allergic asthma both in a mouse model and in humans [37, 38]. In eosinophilic paranasal mucosa cell culture, IL-13 acts by increasing the levels of beta-catenin, which contributes to cell-cell adhesion in CRS [39]. In a mouse model, treatment with IL-16 systemically diminished the release of IL-5 and bronchoalveolar lavage eosinophilia [40]. However, Lackner et al. has shown the expression levels of serum IL-16, IL-16 mRNA, and IL-16 protein in mucus and tissue specimens and their association with the presence of eosinophils in the nasal polyps of ECRS patients. Finally, IL-16 stimulates the migration and persistence of activated eosinophils in ECRS [41]. The initiating mediators of T-helper 2 (Th2) inflammation are often seen in ECRS; IL-25 and IL-33 are involved in the initiation of Th2 inflammation and eosinophilia. Recent studies showed the overexpression of IL-25 and IL-33 in eosinophilic CRS, suggesting that the release of these cytokines may preserve eosinophilic inflammation in CRS [42, 43]. Elevated levels of serum eosinophils correlate with IL-31 levels in patients with allergic rhinitis and allergic asthma [44]. Moreover, IL-31 together with other cytokines upregulate mucin gene expression and mucus production [2, 45–47], which is the most common symptom of ECRS and contributes to the worsening of the clinical features of the disease.

**Fig. 1:**
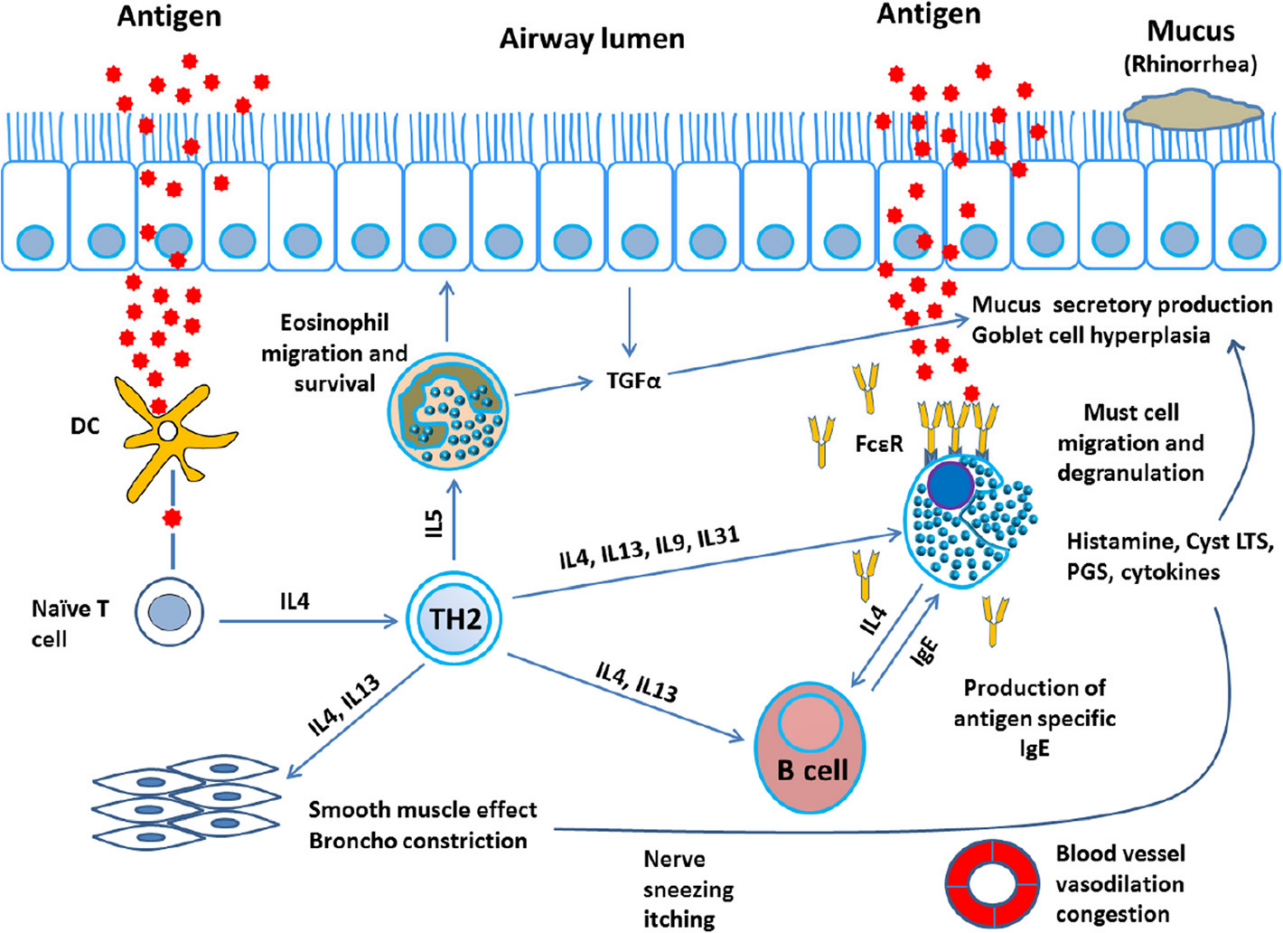
Possible involvement of cytokines, mucus production, and other mediators in the pathogenesis of ECRS

**Fig. 2:**
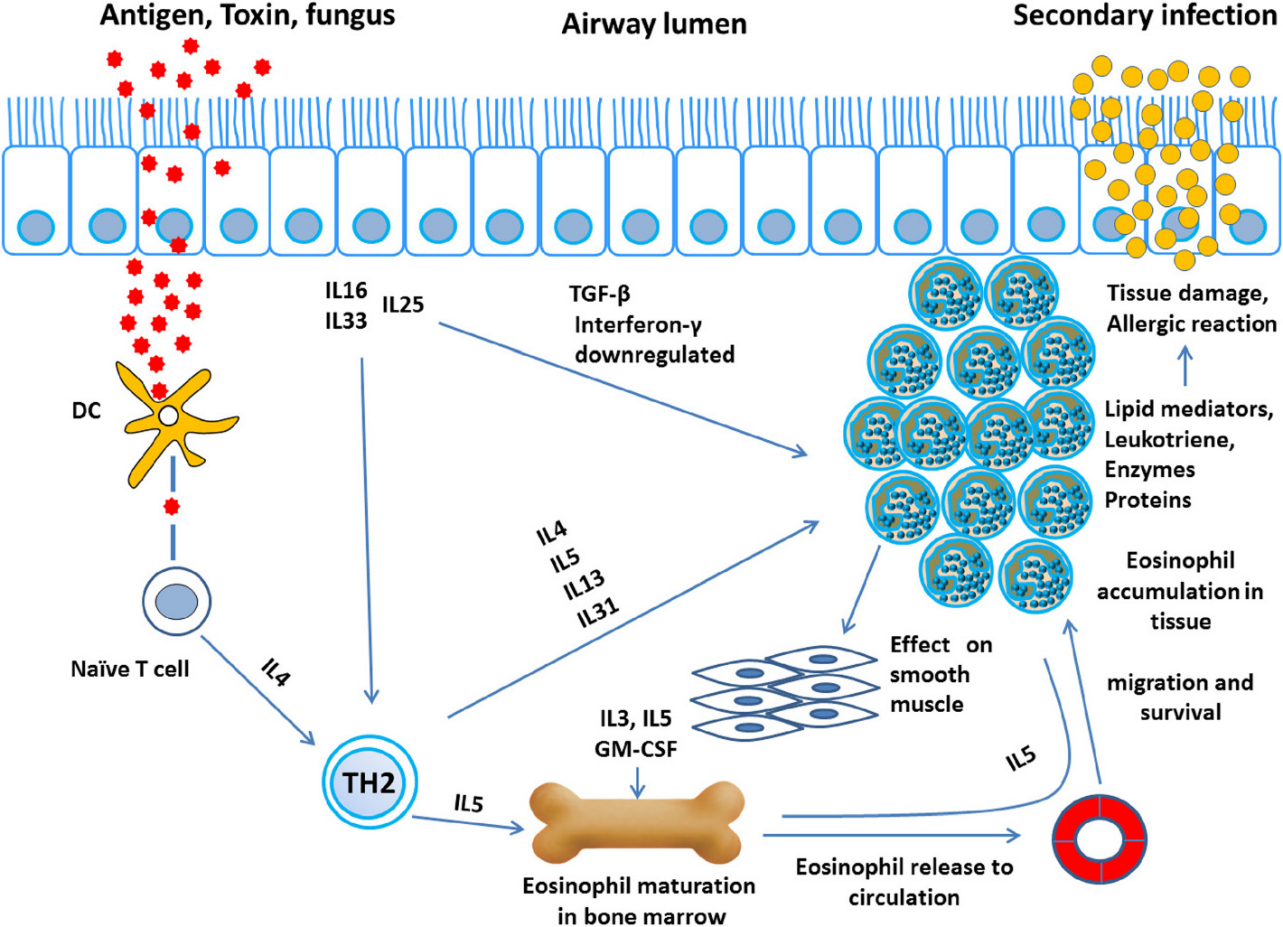
Involvement of cytokines in the production and survival of eosinophils

Conversely, cytokines that have antagonistic effects, especially on Th2 inflammation, such as IFN-γ and transforming growth factor (TGF)-β, are down-regulated in ECRSwNP [48]. The complex chain of cytokines, chemokines, and eosinophils and the mediators secreted by eosinophils are integral to the pathogenesis of ECRS. Therefore, it is important to understand the potential function and activities of these cytokines, chemokines, and eosinophils and the products of eosinophils. Table 1 summarizes the function of cytokines in ECRS, and Figs. 1 and 2 describe the complex pathways of cytokines, mediators, and the survival of eosinophils in the pathogenesis of ECRS.

**Table 1:** Possible functions and activities of cytokines in ECRS

IL-3, IL-5, GM-CSF	Hematopoietic cell development, differentiation, and maturation
IL-4, IL-5, IL-1β, PDGFα	Critical for PDGFRα
IL-9	Regulates granulocytopoiesis in allergic inflammation
IL-13	Increases the levels of beta-catenin, which contributes to cell-cell adhesion, increases mucus production
IL-16	Stimulates the migration of persistently activated eosinophils
TGFα, IL-31, Th2 cytokines	Increase mucus production
IL-25, IL-33	Initiate Th2 inflammation and eosinophilia
IFN-γ, TGF-β	Downregulated in ECRS

### Blood eosinophils

A number of studies demonstrated that there is an association between peripheral eosinophilia and a high percentage of eosinophil infiltration in paranasal sinuses [49]. Eosinophils are considered the most important inflammatory cells in this disease [50]. The accumulation of activated eosinophils within tissue is thought to be a hallmark of this condition [51]. In addition, there is reportedly an association between the eosinophil count and the severity of paranasal cavity lesions in patients with ECRS. Moreover, there is a correlation between an increase in the circulating eosinophil count and the severity of paranasal cavity computed tomography (CT) findings [52]. Furthermore, the percentage of circulatory eosinophils and prevalence of asthma complications are reportedly significantly higher in patients with ECRS than in non-ECRS patients, and are associated with the severity of paranasal cavity lesions [53]. Since blood eosinophilia is significantly correlated with eosinophil infiltration in the nasal polyps of ECRS patients [14], the percentage of blood eosinophils could be a good marker for eosinophilic inflammation of the nasal polyps [8].

### CT scan images

**Fig. 3:**
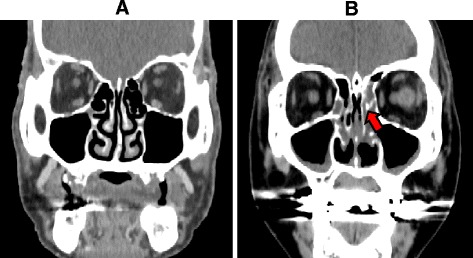
CT images of a normal 75-year-old man (**a**) and a 45-year-old woman diagnosed with ECRS (**b**). The *arrow* indicates opacity in the ethmoid sinuses

A better understanding of the clinical features and specific characteristics of CT images will facilitate the diagnosis of ECRS. Researchers have found a direct association between the severity of paranasal cavity CT findings and an increase in the circulatory eosinophil count [52]. Thus, a blood examination for detecting eosinophils may help with CT findings. Moreover, there is significant correlation between the degree of eosinophil infiltration of the ethmoidal mucosa and the severity of CT scan images [54]. In ECRS, the ethmoid sinuses show predominant opacification, especially in the posterior sinus and olfactory cleft in the early stages, whereas in non-ECRS, maxillary sinuses show predominant opacification in the late stages [14]. Furthermore, for the extent of grading the severity of the disease, the Lund–Mackay staging system (where 0 means no abnormality, 1 means partial opacification, and 2 means total opacification) [55] provides a useful clinical tool. Figure 3a shows a normal CT scan of a 75-year-old man and Fig. 3b shows opacity in the ethmoid sinus of a 45-year-old woman diagnosed with ECRS.

### Diagnosis

Patients with ECRS exhibit clinical characteristics that include long term nasal congestion, mucus production, olfactory disturbances, bilateral nasal polyposis, and intermittent acute exacerbation of secondary bacterial infections [2]. A combination of the cut-off values for three predictors (increased blood eosinophil percentage above the normal range, olfactory cleft score ≥1, and posterior ethmoid score ≥1) has high diagnostic accuracy and can differentiate ECRS from non-ECRS with high accuracy [8]. In ECRS, nasal polyps arise bilaterally from the middle meatus and from inside the middle turbinate, which may be why ECRS patients complain of a smell disorder in the early stage of the illness [14]. In case of allergic fungal sinusitis, there is allergic mucin-type CRS in which the mucus contains clusters of eosinophils, and fungi are also detected by histological examination or culture [56]. Briefly, bilateral nasal polyposis, predominant opacification of the ethmoid sinuses, peripheral blood eosinophil count above the normal range, strong tendency for and recurrence of nasal polyps after surgery, and effectiveness of systemic steroids against recurrent nasal polyps together with the characteristic clinical signs and symptoms of the disease confirm the diagnosis [8, 57]. Therefore, it seems that detailed present and past history/physical examinations of the patient, response to previous treatments, laboratory investigations, manifestation of olfactory dysfunction, and CT scan would be helpful for the diagnosis of ECRS.

### Surgery

Since there is no approved medication to treat patients with ECRSwNP completely, surgery is often needed to clear the sinonasal passage, and repeated endoscopic sinus surgery (ESS) is often required [58]. In cases of chronic sinusitis with severe eosinophilic infiltration, the post-operative prognosis is poor compared to cases with sinusitis where the problem is primarily due to the obstruction of the ostiomeatal complex (OMC) (OMC is the functional unit of the anterior ethmoid complex and provides final common pathway for drainage and ventilation of the frontal, maxillary and anterior ethmoid sinuses) [51, 59, 60]. Therefore, morphological abnormities of the OMC are not considered to have a significant role in sinusitis where there is a high percentage of eosinophil infiltration in the paranasal mucosa. Activated eosinophils in nasal polyps serve as an index in ECRS [61]. Since ECRS is a type of chronic sinusitis that is considered to occur secondarily to systemic eosinophil deregulation [3], the benefit of surgery is significantly less in these patients, especially where the circulating eosinophils represent 6 % or higher of the total number of blood cells. Thus, a high circulating eosinophil count can be considered as an index of poor prognosis [62].

Moreover, the presence of mucosal eosinophilia (>10 eosinophils/high-power field) at the time of ESS consistently predicts less improvement in both disease-specific measures and general quality of life compared to the absence of eosinophilia [63]. Additionally, in a study group, 13 of 14 patients (92.9 %) who were treated with multiple courses of oral corticosteroids, revision surgery, or revision surgery together with oral corticosteroids, showed recurrence after 6 months’ follow-up [11]. Furthermore, surgery is often complicated by adhesions and scarring that can comprise the success of the procedure and the results are often poor [64]. The combination of ESS together with long-term low-dose macrolide therapy relatively controls the symptoms of patients with non-ECRS [65, 66], whereas ECRS is unresponsive to macrolide therapy [8]. In a recent report, the benefit of ESS was shown for both types of CRS, especially for ECRS patients with asthma [67]. Surgery is thought to reduce the need for medication in asthmatic patients [68]. The reduction or complete loss of the sense of smell is a characteristic symptom of ECRS [6, 15, 69], and it was shown that the degree of olfactory dysfunction in sinusitis complicated by asthma, which is a representative disease of eosinophilic infiltration, was more severe than the olfactory dysfunction seen in sinusitis caused by OMC accumulation. However, after ESS, the improvement of patients in whom olfactory dysfunction was caused by asthma was better compared with those in whom olfactory dysfunction was caused by OMC [70]. In a recent study, Lind et al. indicated that there was a significant impact of surgery on patients who had CRSwNP and also CRSsNP [71]. In addition, Costa et al. clarified that patients with recurrent acute rhinosinusitis benefit from both medical and surgical therapeutic interventions [72]. Undoubtedly, there is still the need for further investigations of additional advanced strategies to treat patients with ECRS.

### Medical treatment

The effectiveness of low-dose, long-term erythromycin treatment (macrolide therapy) was reported for the treatment of CRS in Japan [65, 73, 74]. In the 1990s, the combination of macrolide therapy and ESS became the gold standard treatment for CRS [66]. Unfortunately, some CRSwNP cases are refractory to combined medical and surgical interventions. The histological characteristics of these cases showed marked eosinophil infiltration of the nasal polyps [14, 75]. Thus, in Japan, since 2001, the term ECRS has been used to classify this subtype of CRSwNP [6]. The typical symptoms, co-morbid asthma, effectiveness of steroid therapy, and recurrence rate after surgery, clinically differentiate ECRS from CRSwNP (non-ECRS) [6, 14].

As mentioned before, the treatment strategies for ECRS differ from those for non-ECRS; a diagnostic criterion for ECRS is thought to be very useful to decide a treatment strategy in an outpatient setting [8]. If sinusitis with severe eosinophilic infiltration is diagnosed prior to surgery, it would be possible postoperatively to decide specific medication for the treatment of asthma that also has an effect on nasal polyposis, instead of macrolides. Additionally, it would be possible to make it clear to patients with sinusitis involving eosinophils that they have a high possibility of a poor postoperative prognosis that will require long-term postoperative care [69].

Systemic deregulation of eosinophils is thought to be involved in the pathogenesis of ECRS [3]. Once the number of eosinophils increases in the circulatory system, they accumulate rapidly in tissues where they secrete enzymes and proteins that can damage tissues, and they also synthesize and release lipid mediators that can cause edema, bronchoconstriction, and chemotaxis [22]. Eosinophils are therefore an ideal target for the selective inhibition of tissue damage [29]. IL-5 is required for the maturation of eosinophils in bone marrow and their release into the blood [24–26]. Both in humans and animals, inhibiting IL-5 with monoclonal antibodies (mAbs) can decrease circulatory and bronchoalveolar eosinophilia caused by an allergic challenge or chronic disease [76–79]. Anti-IL-5 antibodies inhibit the action of IL-5, which has an important role in the pathogenesis of asthma by damaging tissue due to eosinophil accumulation during pulmonary inflammation. Recently, multiple studies have shown the efficacy of mepolizumab, a humanized anti-IL-5 mAb that is considered to be safe and has a significant effect on the recovery of nasal polyposis characterized by eosinophilic inflammation [80, 81]. Moreover, it does not induce immunosuppressive consequences that can arise from the systemic use of drugs such as steroids [29].

Although, CRSwNP is characterized histologically by an abundance of eosinophilic inflammatory changes, the levels of leukotriene C4 in patients with recurrent sinonasal polyps after surgery are significantly higher than in healthy controls, and higher leukotriene C4 levels can be an indicator of the risk of recurrence of nasal polyps [82]. Recent studies have shown the role of leukotrienes in asthma and chronic sinusitis, specifically in those subtypes involving eosinophils. Leukotriene receptor antagonists, for example, montelukast and zafirlukast, and the leukotriene synthesis inhibitor zileuton have been shown to reduce the symptoms and decrease the steroid requirement of these patients [83, 84].

In European [85] and United States [86] guidelines, corticosteroids are the first-line and most effective treatment for patients with CRSwNP. Basically, intranasal corticosteroids are used as a first-line treatment [87, 88]. Wang et al. showed the efficacy and clinical improvement of short-term intranasal budesonide nebulization in patients with ECRS [89]. However, for cases that are not controlled with intranasal corticosteroids, a short course of oral corticosteroids is required for CRSwNP, and in cases where medical therapy has failed, ESS is required [87, 88]. The current guidelines for severe CRSwNP patients also recommend combined oral and intranasal corticosteroid therapeutic strategies. Steroids modulate nasal polyp mucosa remodeling, particularly by promoting epithelial repair and regulating tissue remodeling markers, increase total collagen content, reduce tissue eosinophil infiltration [90], improve nasal symptoms and airflow, and reduce the size of polyps [91]. The mechanism underlying the effects of nasal steroids seems to be multifactorial, starting with their binding to glucocorticoid receptors, which reduces the number and degree of antigen-presenting cells, activated T cells, and eosinophils [49]. The cellular mechanism that induces cell resistance to topical glucocorticoids may be one of the major causes of the clinical failure of ECRS treatment [92, 93].

Alessandri et al. showed that AT7519, a novel cyclin-dependent kinase inhibitor, causes apoptosis in human and mouse model eosinophils, indicating that such inhibitors can have a therapeutic role in eosinophil-dominant allergic disorder treatment [94]. A newly published study clarified that an anti-CD30 mAb significantly increased eosinophil apoptosis compared with controls. By western blot analysis, the anti-CD30 mAb was shown to decrease significantly the expression of Bcl-2 and procaspase-9 and -3 and increased the expression of caspase-9 and -3, suggesting that this mAb induces human eosinophil apoptosis via the Bcl-2 and caspase pathways [95]. In another study, a complex topical stimulus (allergen challenge) was applied to the tracheobronchial airway of guinea pig, and eosinophils were determined by selective tracheobronchial lavage and histological examination of the tissue. After 10 min, migration of eosinophils into the airway lumen occurred and the numbers of tissue eosinophilia were reduced by 63 and 73 % [96]. A recent study showed that verapamil modulates IL-5 and IL-6 secretion in human sinonasal polyps and may have a possible role in the management of CRSwNP [97]. There are also other multiple studies performed on animal models that have potential use for the treatment of ECRS. Recently, new studies have shown the role of the effects of intranasal cyclosporine and resveratrol in the management of eosinophilic rhinosinusitis using animal models [98, 99]. Hopefully, these new findings will help to discover advanced therapeutic agents for the treatment of patients suffering from ECRS, and to improve their quality of life.

## Conclusion

ECRS is an intractable and persistent disease of the nose and paranasal sinuses. Eosinophilic degradation and accumulation, the release of cytokines and chemokines, and mucus production have an important role in the pathogenesis of this disease. The clinical features reflect the complicated condition of ECRS that seems to be resistant to the current available medical therapies and has a high rate of recurrence after surgery. Further studies are needed particularly focusing on the function of cytokines, chemokines, and eosinophils and the production of eosinophils to achieve a precise treatment, prevention, and the required outcomes.

## Abbreviations

CRS: chronic rhinosinusitis
CT: computed tomography
ECRS: eosinophilic chronic rhinosinusitis
ECRSsNP: eosinophilic chronic rhinosinusitis without nasal polyps
ECRSwNP: eosinophilic chronic rhinosinusitis with nasal polyps
ESS: endoscopic sinus surgery
GM-CSF: granulocyte-macrophage colony stimulating factor
IFN: interferon
IL: interleukin
mAb: monoclonal antibody
OMC: ostiomeatal complex
PDGFRα: platelet-derived growth factor receptor alpha
PDGFα: platelet-derived growth factor-α
TGF: transforming growth factor
Th2: T-helper 2
